# Pulmonary effects of dexmedetomidine infusion in thoracic aortic surgery under hypothermic circulatory arrest: a randomized placebo-controlled trial

**DOI:** 10.1038/s41598-021-90210-w

**Published:** 2021-05-26

**Authors:** Seongsu Kim, Soo Jung Park, Sang Beom Nam, Suk-Won Song, Yeonseung Han, Sangmin Ko, Young Song

**Affiliations:** 1 Department of Anesthesiology and Pain Medicine, Yonsei University College of Medicine, Seoul, South Korea; 2 Anesthesia and Pain Research Institute, Yonsei University College of Medicine, Seoul, South Korea; 3 Department of Cardiovascular Surgery, Yonsei University College of Medicine, Seoul, South Korea

**Keywords:** Aortic diseases, Aortic diseases

## Abstract

Dexmedetomidine has emerged as a promising organ protective agent. We performed prospective randomized placebo-controlled trial investigating effects of perioperative dexmedetomidine infusion on pulmonary function following thoracic aortic surgery with cardiopulmonary bypass and moderate hypothermic circulatory arrest. Fifty-two patients were randomized to two groups: the dexmedetomidine group received 1 µg/kg of dexmedetomidine over 20 min after induction of anesthesia, followed by 0.5 µg/kg/h infusion until 12 h after aortic cross clamp (ACC)-off, while the control group received the same volume of normal saline. The primary endpoints were oxygenation indices including arterial O_2_ partial pressure (PaO_2_) to alveolar O_2_ partial pressure ratio (a/A ratio), (A–a) O_2_ gradient, PaO_2_/FiO_2_ and lung mechanics including peak inspiratory and plateau pressures and compliances, which were assessed after anesthesia induction, 1 h, 6 h, 12 h, and 24 h after ACC-off. The secondary endpoints were serum biomarkers including interleukin-6, tumor necrosis factor-α, superoxide dismutase, and malondialdehyde (MDA). As a result, dexmedetomidine did not confer protective effects on the lungs, but inhibited elevation of serum MDA level, indicative of anti-oxidative stress property, and improved urine output and lower requirements of vasopressors.

## Introduction

Cardiovascular surgery under cardiopulmonary bypass (CPB) is commonly accompanied by postoperative pulmonary dysfunction, ranging from temporary minor hypoxia to severe fatal manifestations, such as acute respiratory distress syndrome (ARDS)^1^. Regardless of its severity, pulmonary dysfunction impairs patient outcomes and substantially increases the healthcare burden^2^. The risk of it is significantly high in the aortic surgery because of massive inflammation and oxidative stress caused by profound ischemia–reperfusion (IR) after hypothermic circulatory arrest (HCA) and CPB, massive blood transfusion, and surgical damage^3^. Indeed, of the various surgery types, aortic surgery has been identified as a strong independent risk factor for postoperative ARDS occurrence^4^. However, there is currently no protective modality with consistent efficacy and safety.

Dexmedetomidine, a highly selective α_2_ adrenergic agonist that is widely used for sedation and analgesia, has emerged as a promising organ protective agent with its anti-inflammatory and anti-oxidative stress properties^5,6^. Putative mechanisms include inhibition of noradrenaline-overflow and activation of vagus nerve and nicotinic acetylcholine receptor, which are related to suppression of inflammatory cytokines^7–9^. It is also known that α adrenergic system is involved in activation of antioxidant enzymes, which supports anti-oxidative stress property of dexmedetomidine^10^. In patients undergoing cardiac surgery with CPB, intraoperative dexmedetomidine infusion reduced the levels of pro-inflammatory cytokines during and after CPB^11^. Additionally, it was shown to reduce the rate of renal injury in adults^12–14^, pediatric cardiac surgery^15^, and major abdominal surgery^16^. With regard to lung protection, it reduced lung injury in various animal experimental models including mechanical ventilation-induced injury, toxic substances, hemorrhagic shock, sepsis, and IR of lungs and intestine^17–22^. Clinically, intraoperative dexmedetomidine infusion improved the quality of oxygenation and lung mechanics in patients undergoing lung resection surgery^23^. The protective effect was also shown in patients with morbid obesity and restrictive lung disease undergoing bariatric surgery^24^. However, it is not clear whether dexmedetomidine could protect the lungs in cardiovascular surgery settings.

Accordingly, this prospective randomized placebo-controlled trial aimed to investigate the effects of perioperative dexmedetomidine infusion on the oxygenation function and respiratory mechanics following thoracic aortic surgery with CPB and moderate HCA.

## Methods

### Participants

This study was conducted in Gangnam Severance Hospital at the Yonsei University College of Medicine between February 2016 and February 2018 following approval of study protocol by institutional review board and hospital research ethics committee (# 3-2015-0252) and registration at http://clinicaltrials.gov (NCT02678728) on February 10, 2016. Patients aged ≥ 20 years scheduled for proximal thoracic aortic surgery under CPB and moderate HCA were considered eligible for enrollment. Exclusion criteria were the presence of pre-existing pulmonary disease requiring medication, such as bronchodilator or systemic steroids, hemodynamic instability requiring inotropes and/or vasopressors, uncontrolled dysrhythmias, left ventricular ejection fraction (LVEF) < 30%, illiteracy, and pregnancy. Written informed consent was obtained from all the study participants.

### Perioperative management 

All methods of this study were performed in accordance with the institution guidelines and regulations^25^. Briefly, monitoring during surgery included thermodilutional pulmonary artery catheter use, the measurement of bilateral radial arterial pressure, the calculation of the bispectral index (BIS, Medtronic, Dublin, Ireland), and the use of transesophageal echocardiography, cerebral oximetry (NIRS, INVOS 4100; Covidien, Mansfield, MA), and esophageal and rectal temperature probes. Anesthesia was induced using midazolam, sufentanil, and rocuronium bromide and maintained with sevoflurane and the continuous infusion of remifentanil. The right axillary artery and left femoral artery were cannulated for CPB. After sternotomy, non-pulsatile pump flow was conducted at a rate of 2.0–2.4 L/min/m^2^ under mild hypothermia (32–34 °C). Upon reaching a target rectal temperature of 28 °C, circulatory arrest to aid distal anastomosis was initiated while the antegrade cerebral perfusion via right axillary artery was maintained at a pressure of 60 mmHg. If the level of cerebral oxygen saturation during HCA decreased by 20% of the baseline value or lower, bilateral cerebral perfusion was implemented. Lower body perfusion was performed through the femoral artery at a rate of 0.8–1.0 L/min/m^2^ for 1 min every 15 min. After distal anastomosis, the proximal graft was cross-clamped (aortic cross clamp, ACC) and the left head vessels and proximal aorta were reconstructed. After ACC release (ACC-off), the innominate artery was reconstructed, followed by separation from CPB. During surgery, the mean arterial pressure (MAP) was maintained at > 60 mmHg and < 80 mmHg using norepinephrine, vasopressin (in the case of norepinephrine > 0.3 μg/kg/min), and nicardipine. Milrinone was infused when pulmonary hypertension or ventricular dysfunction persisted after ACC-off. Fluid resuscitation was performed with 6–8 mL/kg crystalloid solution (Plasma Solution-A Injection1000 mL; CJ Parma, Seoul, South Korea). Fresh frozen plasma and platelets were transfused immediately following weaning from CPB at a level of 390–650 mL and 240–480 mL, respectively, according to the patients’ body weight and bleeding pattern. Packed red blood cells were administered if the hematocrit value was < 20% during CPB and < 24% otherwise. The same protocols, in terms of hemodynamic management and transfusion, were applied during the intensive care unit (ICU) admission.

### Study protocol

The study participants were randomly assigned to either the control group or dexmedetomidine group at a 1:1 ratio according to a computerized randomization table created by an independent investigator, who then prepared 4 µg/ml of dexmedetomidine (Precedex, Hospira, Lake Forest, Illinois, USA) for the dexmedetomidine group or same volume of 0.9% normal saline for the control group in identical 50-ml syringes. Group allocation was concealed by opaque and sequentially numbered envelopes. Which were opened just before surgery by the attending anesthesiologist. The study drugs were administered intravenously by the attending anesthesiologist after the induction of anesthesia by the loading of 1 µg/kg over 20 min followed by a 0.5 µg/kg/h infusion until 12 h after ACC-off, which was based on previous studies on cardiac surgery^26^, with some consideration of safety issues on hemodynamics and sedative effect. The lungs were ventilated after the induction of anesthesia using autoflow volume-controlled ventilation (Primus™, Draeger AG & Co. KGaA, Lübeck, Germany) with a tidal volume of 8 mL/kg of predicted body weight and positive end-expiratory pressure (PEEP) of 5 mmHg at an inspiratory: expiratory ratio of 1:2. The initial fractional inspired oxygen (FiO_2_) level was set at 0.5 and the respiratory rate was adjusted for the maintenance of an end-tidal CO_2_ level of 30–40 mmHg. When a full CPB flow was achieved, mechanical ventilation was stopped while a continuous positive airway pressure of 5 cmH_2_O was applied. After weaning from CPB, the lungs were recruited manually at 40 cmH_2_O for 8 s and then the pre-CPB mechanical ventilation strategy was applied. If the arterial O_2_ partial pressure to FiO_2_ (PaO_2_/FiO_2_) ratio was lower than 300 mmHg, the PEEP and FiO_2_ values were raised in a step-by-step manner, according to the National Institutes of Health ARDS Clinical Network protocol. Extubation was conducted at the ICU in accordance with the standard care guidelines by the attending physician who was blinded to the group assignment. The study drugs were discontinued in case of persistent hypotension (MAP < 60 mmHg) or bradycardia (heart rate [HR] < 50 bpm) in spite of the provision of proper fluid resuscitation and cardiovascular drug therapy, and resumed after recovery. The patients, surgeons, attending anesthesiologists, ICU physicians, and outcome assessors were all blinded to the group allocation throughout the study. 

### Study endpoints

The primary endpoints were the oxygenation indicators, including the arterial O_2_ partial pressure to alveolar O_2_ partial pressure ratio (a/A ratio), alveolar-arterial (A–a) O_2_ gradient, and PaO_2_/FiO_2_ as well as the indicators of lung mechanics, including dynamic compliance, static compliance, peak inspiratory pressure, and plateau pressure, all of which were assessed after the induction of anesthesia, 1 h, 6 h, 12 h, and 24 h after ACC-off. These data were obtained from the arterial blood gas analysis and ventilator-derived parameters.

The secondary endpoints were serum biomarkers of inflammation and oxidative stress, including interleukin (IL)-6, tumor necrosis factor (TNF)-α, superoxide dismutase (SOD), and malondialdehyde (MDA), as measured with the Human IL-6 Quantikine ELISA kit, Human TNF-alpha Quantikine HS ELISA kit (R&D System Inc., Minneapolis, MN, USA), Human SOD ELISA Kit, and Human MDA ELISA Kit (MyBioSource, SD, CA, USA), respectively, at the same time points as the primary endpoint assessment. Postoperative 30-day morbidity endpoints, including ARDS, which was defined as acute onset hypoxemic respiratory insufficiency with PaO_2_/FiO_2_ < 300 mm Hg in the presence of bilateral pulmonary infiltrates and the absence of left atrial hypertension^27^, myocardial infarction, new-onset atrial fibrillation, delirium, cerebrovascular attack, gastrointestinal tract ischemia or hemorrhage, acute kidney injury (AKI)^28^, renal replacement therapy, deep sternal wound infection, mortality, and length of ICU and hospital stay were also evaluated. 

### Other assessments

The patients’ demographic data, including history of hypertension, diabetes mellitus, chronic kidney disease, cerebrovascular accidents, coronary artery disease, cardiovascular medications, Euroscore, and LVEF were recorded. Preoperative pulmonary function test comprising forced vital capacity, forced expiratory volume in one second, and carbon monoxide diffusion capacity was recorded in elective cases only, because patients who received emergency surgery were not able to perform the test.

The perioperative variables included aortic pathology, emergency surgery, operation time, total CPB time, ACC time, HCA time, intraoperative and postoperative 24 h fluid intake, urine output, blood loss, transfusion, and the need for vasopressor and inotropic agents.

### Statistical analysis

Study sample size was calculated using the PASS 12 (NCSS, LLC, Utah, USA). In previous studies on pulmonary protection against IR during surgery, their interventions resulted significant improvement in the a/A O_2_; 0.067^29^ and 0.23^30^, which were related to better clinical outcomes. We assumed that improvement of a/A O_2_ within this range would reflect therapeutic efficacy of study drug. In our past record of aortic surgery, the a/A O_2_ at 12 h after ACC-off was 0.4 ± 0.15. With the expectation of an improvement of 0.1 in the a/A O_2_ by dexmedetomidine, the sample size calculation for 5 times repeated measures design revealed that 22 patients would be required in each group for the obtainment of a power of 80% at an alpha value of 0.05. Assuming a 20% dropout rate, we decided to enroll 52 patients. 

Data were analyzed using SAS software 9.3 (SAS Inc., Cary, NC, USA) and SPSS version 23 (SPSS Inc., Chicago, IL, USA). After normality test using the Shapiro–Wilk test, the continuous variables were compared between the groups using an independent t-test or a Mann–Whitney U test and expressed as the mean ± standard deviation or median [interquartile range]. Categorical variables were compared between the groups using the chi-square or Fisher’s exact test and expressed as the number of patients (%). Serially measured variables, including the indicators of oxygenation, lung mechanics, serum biomarkers, and hemodynamic data were analyzed using linear mixed models (LMMs), with group, time, and group-by-time as fixed effects. A *P* value < 0.05 was considered statistically significant.

## Results

**Figure 1:**
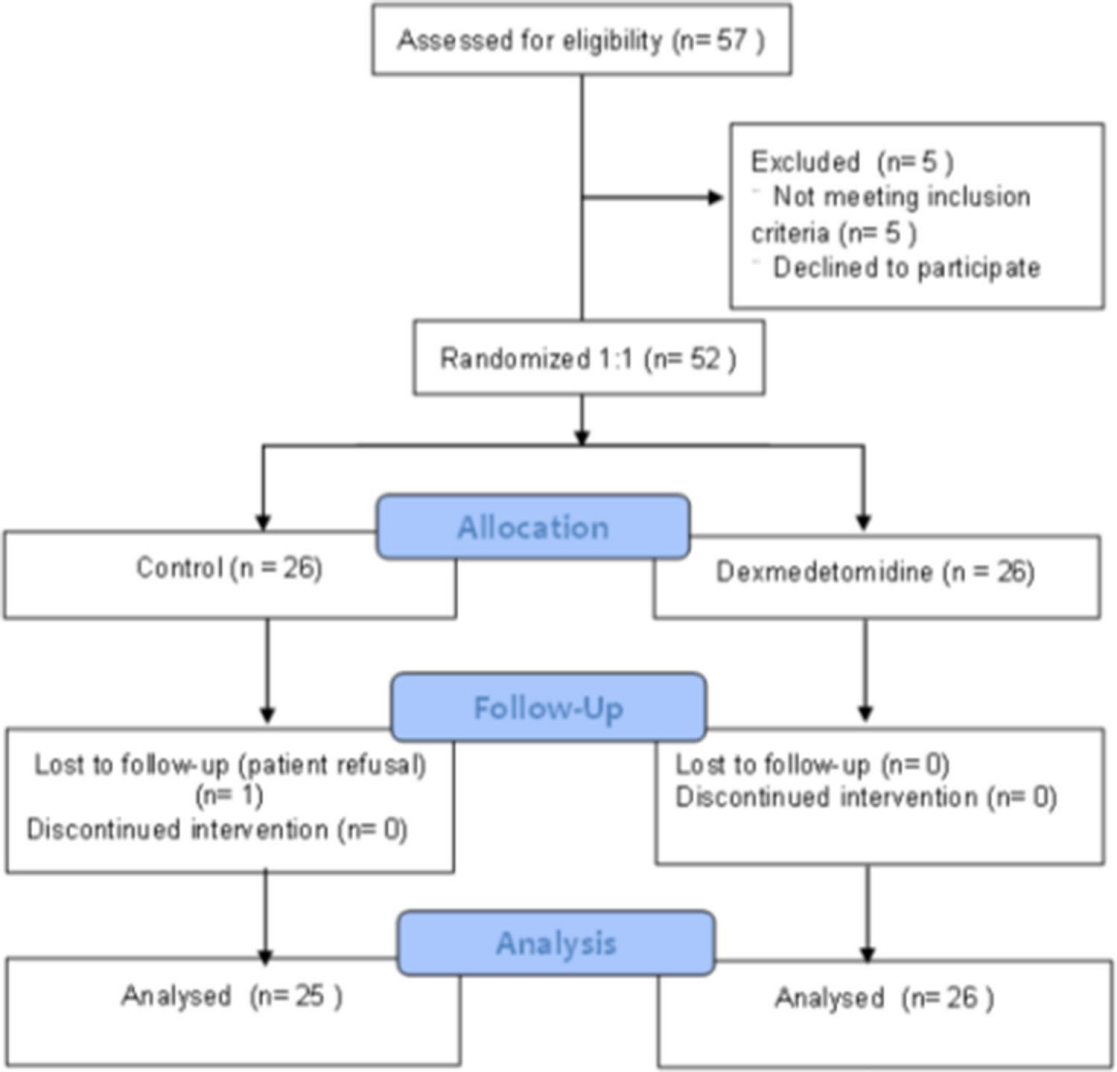
Flow chart showing the patient enrollment process based on CONSORT guidelines.

Of the 57 patients assessed for eligibility, 52 met the inclusion criteria and provided informed consent. One patient who withdrew consent after randomization was excluded, and a total of 51 patients were included in the analysis (Fig. 1). None developed serious adverse events related to administration of study drugs.

Patient demographics, pre-existing chronic disease and medication use, and preoperative cardiac and pulmonary function were not different between the groups (Table 1).

**Table 1:** Preoperative clinical characteristics.

Parameters	Control group (n = 25)	Dexmedetomidine group (n = 26)	*P* value
Age (years)	61 ± 17	62 ± 17	0.838
Sex, male/female	14 /11	14/12	0.877
Height (cm)	165.7 ± 10.4	165.9 ± 10.4	0.937
Weight (kg)	64.5 ± 14.3	69.7 ± 15.2	0.215
Hypertension	17 (68.0)	18 (69.2)	0.925
Diabetes mellitus	3 (12.0)	3 (11.5)	> 0.999
Chronic kidney disease	2 (8.0)	2 (7.7)	> 0.999
Cerebrovascular accident	2 (8.0)	2 (7.7)	> 0.999
Coronary artery disease	4 (16.0)	3 (11.5)	0.703
**Medication**			
β-Blockers	7 (28.0)	5 (19.2)	0.461
Calcium channel blockers	11 (44.0)	8 (30.8)	0.329
RAS blockers	9 (36.0)	12 (46.2)	0.461
Diuretics	4 (16.0)	2 (7.7)	0.419
Preoperative pulmonary function***			
FVC	96.1 ± 25.5	93.4 ± 16.2	0.814
FEV1	103.6 ± 24.3	96.4 ± 14.1	0.504
FEV1/FVC	73.3 ± 3.5	70.1 ± 8.3	0.382
DLCO	90.9 ± 17.0	90.83 ± 10.3	0.998
Euroscore	6 ± 5	7 ± 7	0.880
Preoperative LVEF (%)	62 ± 9	64 ± 6	0.538

Values are shown as the number of patients, mean ± standard deviation.

*DLCO* carbon monoxide diffusion capacity, *FEV1* forced expiratory volume in one second, *FVC* forced vital capacity, *LVEF* left ventricular ejection fraction, *RAS* renin-angiotensin system.

***This data was obtained from patients who received elective surgery.

Operative data including the type of aortic pathology, emergency surgery, duration of surgery, CPB, ACC, and HCA were similar between the groups (Table 2). The degree of intraoperative fluid intake, and volume of transfusion were not different between the groups, but the urine output was significantly higher in the dexmedetomidine group than that in the control group (1435 [1165–1840] vs. 1070 [600–1330] mL; *P* = 0.005). In addition, the amount of norepinephrine administered during the surgery was significantly lower in the dexmedetomidine group than that in the control group (172.0 [40.0–344.0] vs. 360.0 [170.0–695.0] μg; *P* = 0.027). The postoperative 24 h fluid balance was comparable between the groups. The number of patients requiring norepinephrine during the postoperative 24 h period was similar between the groups, whereas the amount of nicardipine during this period was significantly lower in the dexmedetomidine group than that in the control group (324.0 [100.1–820.9] vs. 796.1 [361.7–1591.0] μg; *P* = 0.026).

**Table 2:** Perioperative data.

Parameters	Control group (n = 25)	Dexmedetomidine group (n = 26)	*P* value
Aneurysm/dissection/IMH or PAU	10 (40)/14 (57)/1 (4)	7 (27)/16 (62)/3 (12)	0.428
Valve replacement	1 (4.0)	2 (7.7)	> 0.999
Aortic root replacement	1 (4.0)	1 (3.9)	> 0.999
Emergency	15 (60)	18 (69.23)	0.491
Operation time (min)	298 ± 63	311 ± 77	0.509
Total CPB time (min)	170 ± 48	174 ± 45	0.760
ACC time (min)	71 ± 41	83 ± 49	0.377
HCA time (min)	50 ± 14	51 ± 14	0.820
**Intraoperative input and output**			
Crystalloid input (mL)	2330 (1675–2700)	1900 (1400–2425)	0.214
Urine output (mL)	1070 (600–1330)	1435 (1165–1840)	0.005*
Cell saver transfused (mL)	850 (733–1022)	1000 (300–1245)	0.627
pRBCs transfused (mL)	250 (0–750)	480 (0–750)	1
FFP transfused (mL)	733 ± 186	765 ± 190	0.545
Platelet transfused (mL)	576 ± 177	560 ± 204	0.780
**Intraoperative cardiovascular drugs**			
Amount of norepinephrine (μg)	360.0 (170.0–695.0)	172.0 (40.0–344.0)	0.027*
Patients requiring vasopressin	6 (24.0)	7 (26.9)	0.811
Patients requiring milrinone	3 (12.0)	5 (19.2)	0.478
Amount of nicardipine (μg)	0.0 (0.0–200)	200.0 (0.0–1900)	0.081
**Postoperative 24 h input and output (mL)**			
Crystalloid input (mL)	4295 ± 1107	4046 ± 1178	0.441
Urine output (mL)	3977 ± 1516	3706 ± 1222	0.484
Cell saver transfused (mL)	0 (0–0)	0 (0–213)	0.183
pRBCs transfused (mL)	0 (0–207)	0 (0–0)	0.543
FFP transfused, mL	0 (0–575)	0 (0–215)	0.204
Platelet transfused (mL)	0 (0–0)	0 (0–0)	1
Blood loss (mL)	628 (495–887)	640 (419–844)	0.836
**Postoperative 24 h cardiovascular drugs**			
Patients requiring norepinephrine	11 (44.0)	12 (46.2)	1.000
Amount of nicardipine (μg)	796.0 (362.0–1591.0)	324.0 (100.0–821.0)	0.026*

Values are shown in number of patients(percentage), mean ± standard deviation or median (interquartile range). *P value < 0.05. *ACC* aortic cross clamp, *CPB* cardiopulmonary bypass, *FFP* fresh frozen plasma, *HCA* hypothermic circulatory arrest, *IMH* intramural hematoma, *PAU* penetrating atherosclerotic ulcer, *pRBC* packed red blood cell.

**Figure 2:**
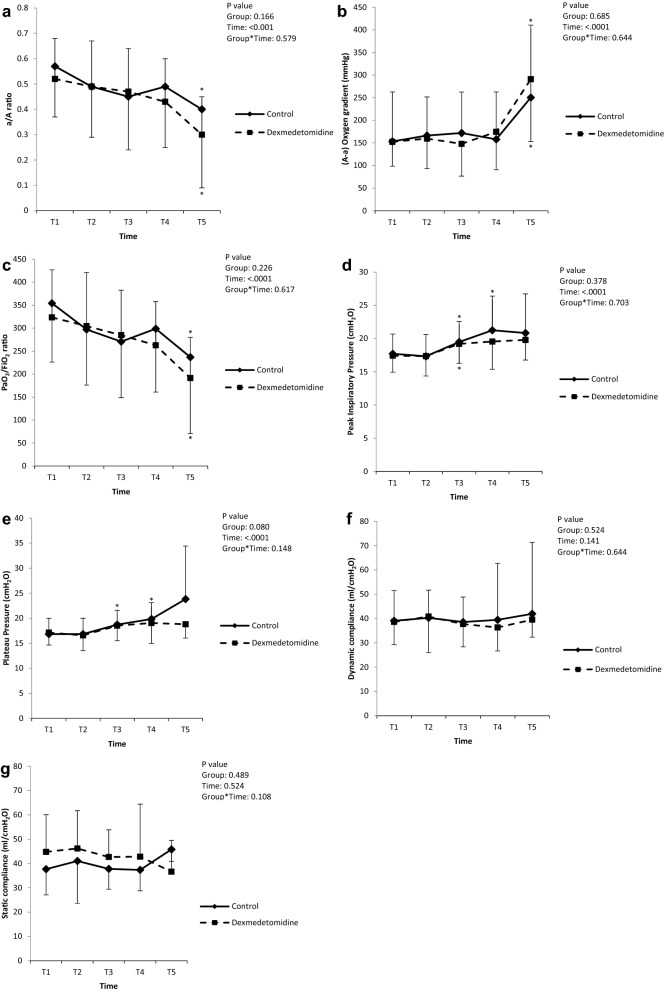
Changes in the lung function indices. a/A, arterial O_2_ partial pressure to alveolar O_2_ partial pressure ratio; A–a, alveolar-arterial; PaO_2_, partial pressure of oxygen; FiO_2_, fraction of inspired oxygen; T1, post-induction; T2, ACC 1 h; T3, ACC 6 h; T4, ACC 12 h; T5, ACC 24 h. *P < 0.05 versus baseline.

As shown in Fig. 2, the LMM analysis revealed that none of the primary endpoints showed time-group interactions or intergroup differences. In the post-hoc analysis of the intra-group changes over time, the a/A O_2_ was significantly decreased at 24 h after ACC-off compared to the baseline values in both groups (*P* = 0.026 and 0.020 in the control group and dexmedetomidine group, respectively). The (A–a) O_2_ gradient value significantly increased at 24 h after ACC-off compared to the baseline values in both groups (*P* = 0.020 and < 0.001 in the control group and dexmedetomidine group, respectively). The PaO_2_/FiO_2_was also significantly decreased at 24 h after ACC-off in both groups (*P* = 0.004 and < 0.001 in the control group and dexmedetomidine group, respectively) compared to the values at the baseline. The peak inspiratory pressure was significantly increased at 6 h after ACC-off in both groups (*P* = 0.022 and 0.015 in the control and dexmedetomidine group, respectively) and 12 h after ACC-off in the control group (*P* = 0.020) compared to the baseline values. The plateau pressure was significantly increased at 6 h and 12 h after ACC-off in the control group only (*P* = 0.010 and 0.002, respectively). The dynamic and static lung compliance values did not change significantly over time in both groups. Subgroup of chronic aneurysm patients who received non-urgent elective surgery showed no significant improvement by dexmedetomidine in the LMM analysis of lung oxygenation function indices (Supplemental Table 1).

**Figure 3:**
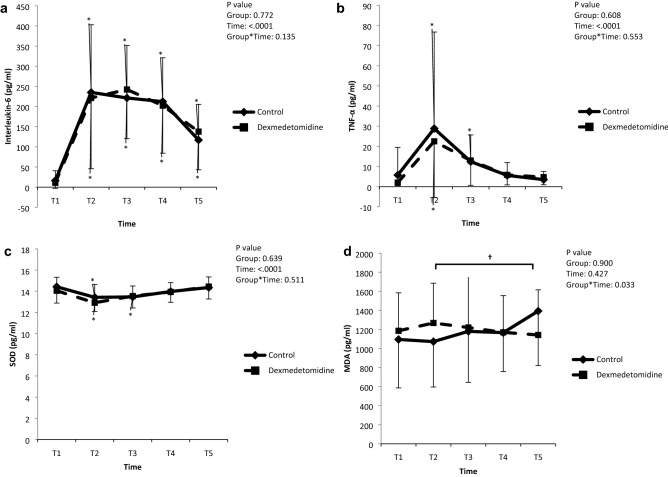
Changes in the levels of biomarkers. TNF, tumor necrosis factor; SOD, superoxide dismutase; MDA, malondialdehyde; T1, post-induction; T2, ACC 1 h; T3, ACC 6 h; T4, ACC 12 h; T5, ACC 24 h. *P < 0.05 versus baseline; ^**†**^time-group interaction.

The LMM analysis on the serum levels of IL-6, TNF-α, and SOD also showed no significant time-group interactions and no intergroup differences (Fig. 3). In the post-hoc analysis of intra-group changes over time, the level of IL-6 was significantly increased at 1 h, 6 h, 12 h, and 24 h after ACC-off compared to the baseline value in both groups (all *P* < 0.001) and the level of TNF-α was significantly increased at 1 h after ACC-off in both groups (*P* = 0.019 and 0.042 in the control group and dexmedetomidine group, respectively) and 6 h after ACC-off in the dexmedetomidine group only (*P* = 0.011) compared with the baseline values. The level of SOD was significantly decreased at 1 h after ACC-off in both groups (*P* < 0.001) and 6 h after ACC-off in the control group only (*P* = 0.002) compared to the baseline values. On the other hand, there were significant time-group interactions in the LMM analysis of MDA level (*P* = 0.033) and the post-hoc analysis revealed that the difference between the groups were in changes between the 1 h and 24 h after ACC-off (*P* = 0.012); it was increased in the control group and decreased in the dexmedetomidine group.

**Figure 4:**
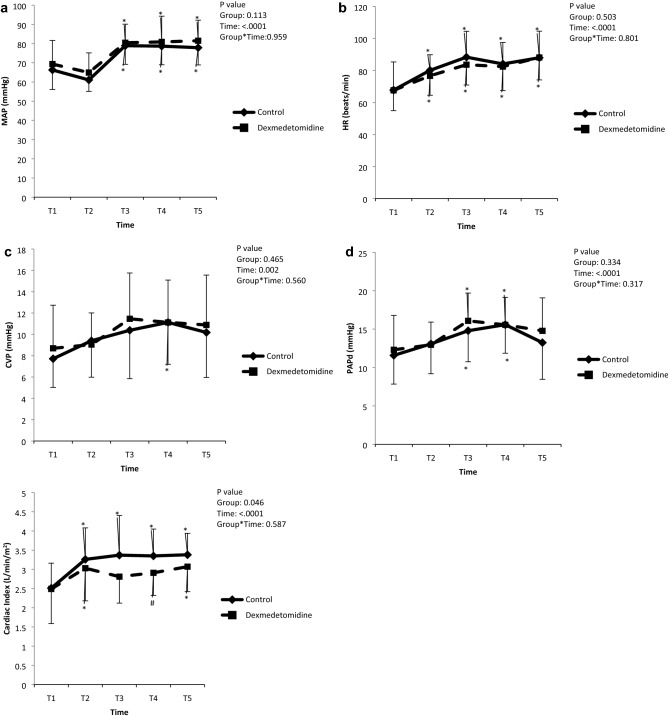
Changes in the perioperative hemodynamic data. MAP, mean arterial pressure; HR, heart rate; CVP, central venous pressure; PAPd, diastolic pulmonary arterial pressure; T1, post-induction; T2, ACC 1 h; T3, ACC 6 h; T4, ACC 12 h; T5, ACC 24 h. *P < 0.05 versus baseline; ^#^*P* < 0.05 versus control.

Changes in the MAP, HR, central venous pressure, diastolic pulmonary arterial pressure, and cardiac index over time were significant in each group (*P* < 0.05 in both group), but there were no time-group interactions in any of the variables, indicating no difference between the groups in hemodynamics (Fig. 4).

The incidence of the postoperative 30-day morbidity endpoint was not statistically different between the groups. Duration of ventilator support at the ICU, need for prolonged ventilator support (> 24 h), need for re-intubation, and lengths of ICU stay and hospital stay were also comparable between the groups. However, there was a trend toward a lower rate of ARDS occurrence in the dexmedetomidine group compared to that in the control group (0 vs. 4 [16%]; *P* = 0.051) (Table 3).

**Table 3:** Early postoperative morbidity.

Parameters	Control group (n = 25)	Dexmedetomidine group (n = 26)	P value
**Major morbidity endpoints in 30 days**			
Myocardial infarction	0	0	
Atrial fibrillation	4 (16.0)	2 (7.7)	0.419
Delirium	9 (36.0)	9 (34.6)	0.918
Cerebrovascular attack	0	1 (4)	> 0.999
ARDS	4 (16.0)	0 (0.0)	0.051
GI tract ischemia or hemorrhage	2 (8.0)	1(3.9)	0.610
Acute kidney injury	7 (28.0)	4 (15.4)	0.274
Renal replacement therapy	3 (12.0)	0 (0.0)	0.110
Deep sternal infection	0	0	
Mortality	2 (8.0)	0 (0.0)	0.235
Ventilator support after surgery (h)	16.00 [10.50–22.50]	16.00 [8.35–21.38]	0.977
Prolonged ventilator support > 24 h	6(24.0)	4 (15.4)	0.499
Re-intubation	1(4.0)	1 (3.9)	> 0.999
Intensive care unit stay (h)	58 (47.0–75.0)	54 (46.8–97.3)	0.985
Hospital stay (day)	18 (12.0–25.0)	15 (11–20.8)	0.565

Values are shown in number of patients (percentage), mean ± standard deviation or median (interquartile range). *ARDS* acute respiratory distress syndrome, *GI* gastrointestinal.

## Discussion

In the present study, administration of dexmedetomidine did not confer protective effects on the lungs in aortic surgery under CPB and HCA. So far, there has been substantial evidence supporting the protective effects of dexmedetomidine on the lungs against various pathological states. In a rat experimental model of IR injury, dexmedetomidine reduced the rate of histological damage to the lungs and inhibited the degree of increase in the levels of inflammatory cytokines including TNF-α and IL-6^22^. In a sepsis model, dexmedetomidine attenuated the level of secondary lung and kidney damage^19^. In clinical studies, the use of dexmedetomidine improved the oxygenation and dynamic compliance in patients with moderate chronic obstructive pulmonary disease (COPD) during lung cancer surgery^23^ and prevented the occurrence of postoperative pulmonary complications following orofacial surgery^31^. Moreover, the intraoperative infusion of dexmedetomidine suppressed the increases in the level of high mobility group box-1 and IL-6 after CPB^11^. Similarly, the infusion of dexmedetomidine in sepsis patients following abdominal surgery attenuated the degree of increase in the levels of TNF-α, IL-1, and IL-6^32^. Therefore, it was assumed that the laboratory and clinical indicators of IR injury of the lungs in patients undergoing aortic surgery would be improved with the use of dexmedetomidine.

However, perioperative infusion of dexmedetomidine in our study neither improved oxygenation and lung mechanics and nor reduced systemic inflammatory insults, which were inconsistent with previous findings^22–24,31^. Of the primary endpoints of our study, the a/A O_2_, which was used for the power calculation, accurately reflects lung oxygenation and is not easily influenced by FiO_2_. So it has long been used in clinical studies. It was significantly improved by effective therapeutic modalities in studies on abdominal aortic surgery and lower extremity IR, and it was related to better clinical outcomes, such as shorter ventilator support^29,30^. Conversely, dexmedetomidine did not improve it, which seems to be associated with the lack of significant benefit on the clinical end points including requirement for ventilator support and the incidence of ARDS as well as mortality. Such an inconsistency between the previous studies and ours may be attributable to greater extent of IR injury, inflammatory reaction, and oxidative stress induced by HCA in addition to CPB and ACC. A serum level of IL-6 at 1 h after ACC-off in our study patients was nearly twice as high as the value measured at the same time point in patients undergoing cardiac surgery without HCA^11^. Although the level of IL-6 was already a little higher at the baseline in our study, which is potentially due to pre-existing greater systemic inflammatory reaction induced by an aortic pathology, the maintenance of circulatory arrest for about 50 min itself could dramatically increase inflammatory response after reperfusion. The impact of dexmedetomidine may be insufficient in overcoming such extensive IR and the consequent lung damage. There was also a similar experimental report that dexmedetomidine treatment did not reduce lung injury induced by systemic IR^17^. More detailed studies focusing on stratified IR injury and the structured dose–response of dexmedetomidine in the clinical settings are clearly warranted.

Previous studies revealed the favorable effects of dexmedetomidine on lung compliance in chronic lung disease patients undergoing surgery. Dexmedetomidine infusion during bariatric surgery in morbidly obese patients decreased the dead space and plateau airway pressure^24^, while its administration during the lung cancer surgery increased dynamic compliance in patients with COPD^23^. We also observed an attenuated increase in the plateau pressure at 6 h and 12 h and peak inspiratory pressure at 12 h after ACC-off with dexmedetomidine use, although there was no significant time-group interaction between the dexmedetomidine group and the control group. Some plausible mechanisms include the inhibitory effects of dexmedetomidine on the airway smooth muscle contraction through the suppression of acetylcholine release and direct action to relax^33^. However, our result should be interpreted carefully, because some positive trend of airway pressure was not connected to the improvement of lung compliances at all.

We observed that dexmedetomidine could inhibit the increase in serum MDA level following reperfusion. Because production of MDA is a result of free radical oxidation of cell membrane phospholipids, serum MDA level is well-established as a biomarker of oxidative stress-induced tissue damage in IR^34^. Anti-oxidative stress property contributes to preventive effect of dexmedetomidine against apoptosis in alveolar epithelial cells^35^, which would be matched to our result. On the post hoc analysis, dexmedetomidine significantly reduced MDA level at 24 h after ACC-off from the value at 1 h after ACC-off (T2), compared with its significant increase in the control group. Although the drug’s impact on changes in MDA from the baseline value (T1) lost statistical significance after calculating *P* values considering repeated measures, continuous decrease of MDA from reperfusion (T2), which contrasts starkly with its surge along the time course in the control group, may indicate potency of dexmedetomidine enough. Nevertheless, it was not linked to significant clinical efficacy, so a careful interpretation is needed. There was a lack of impact of dexmedetomidine on the serum SOD level, which is conflicting with the changes in MDA. Dexmedetomidine could have influenced on other ant-oxidant enzymes, such as catalase and glutathione peroxidase, which warrants further analysis.

Notably, the intraoperative urine output was significantly higher in the dexmedetomidine group than that in the control group, although the incidence of AKI was not different. Protective effects of dexmedetomidine against renal injury in cardiac surgery settings have consistently been reported^36^. A recent randomized controlled trial on the aortic surgery under CPB and HCA also revealed a lower incidence of AKI in patients who received dexmedetomidine for 24 h^13^. The relatively smaller sample size and shorter duration of dexmedetomidine administration in our study may not have allowed for the achievement of statistically significant benefits in terms of the clinical endpoint of renal injury. However, the patients in the dexmedetomidine group required a significantly lower amount of intraoperative norepinephrine and postoperative nicardipine. A large body of evidence states that dexmedetomidine reduces the requirement for exogenous norepinephrine, indicating its ability to preserve blood pressure through improvements of vascular reactivity to catecholamines and angiotensin II as well as its well-known vasoconstrictive property itself^37–39^. Its anesthetic/analgesic-sparing effects may also have contributed to this, although we did not assess those amount^40^. Meanwhile, dexmedetomidine attenuates the level of stress response and suppresses the release of catecholamines in response to a noxious stimulant, which may have stabilized the patients’ postoperative blood pressure, thereby reducing the nicardipine requirement^41,42^. On the other hand, cardiac index was a little lower in the dexmedetomidine group at 12 h after ACC-off, although it was within normal range and the difference was not significant in consideration of time group interaction. This could be attributable to HR-lowering effect and vasoconstrictive properties of dexmedetomidine^39,43^. Although changes of HR was comparable between the groups in our study, dexmedetomidine should be used with caution to avoid critical bradycardia and cardiac collapse^43^.

Limitation of the current study is as follows. Firstly, we calculated sample size with reference to previous studies on surgery associated with IR injury^29,30^. But it could have been insufficient, because IR induced by CPB and total circulatory arrest might be much greater than those of previous reports. Heterogeneity in the disease states and urgency of surgery should have been considered for the sample size determination as well. Secondly, we administered dexmedetomidine for up to 12 h after reperfusion; this duration is significantly shorter than that in previous studies on renal injury^12,13^. As our institutional postoperative recovery protocol includes the extubation at a relatively early time point and discharge from the ICU, we were unable to administer the study drug for longer considering the safety issue. Given the extensive IR injury that occurs upon HCA, a larger amount of dexmedetomidine may have been needed for the inhibition of the inflammatory flare-up and improvement of the lung function.

In conclusions, the administration of dexmedetomidine did not protect the lungs during proximal thoracic aortic surgery under CPB and moderate HCA. Although the levels of pro-inflammatory cytokines were not reduced, the MDA level was attenuated, indicative of the anti-oxidative stress action of dexmedetomidine in aortic surgery. Additionally, we observed improvement of urine output and less use of vasoactive agents during and after surgery by dexmedetomidine treatment.

## Supplementary Information

Supplementary Table.

## Data Availability

The datasets collected and analyzed during this study are available from the corresponding author on reasonable request.
