# Supplementary material for: Pulmonary effects of dexmedetomidine infusion in thoracic aortic surgery under hypothermic circulatory arrest: a randomized placebo-controlled trial
Source: Sci Rep. 2021 May 26;11:10975. doi: 10.1038/s41598-021-90210-w (PMC8155071; doi:10.1038/s41598-021-90210-w)
Supplement: Supplementary file 1 — Supplementary Table. [file 41598_2021_90210_MOESM1_ESM.docx]

| Supplemental Table 1. Linear mixed model analysis of lung function indices in the subgroup of chronic stable aneurysmal patients | | | |
| --- | --- | --- | --- |
| Chronic stable aneurysm | Control group  (n=8) | Dexmedetomidine group  (n=5) | P value |
| a/A O_2_ | | | |
| T1 | 0.633(0.048) | 0.561(0.061) | Group: 0.554  Time: <0.001  Group*Time: 0.491 |
| T2 | 0.545(0.055) | 0.409(0.070) |  |
| T3 | 0.398(0.053) | 0.424(0.067) |  |
| T4 | 0.441(0.054) | 0.479(0.068) |  |
| T5 | 0.284(0.050) | 0.256(0.064) |  |
| (a-A) O_2_ | | | |
| T1 | 123.491(14.026) | 135.720(17.741) | Group: 0.973  Time: 0.004  Group*Time: 0.263 |
| T2 | 140.119(16.858) | 190.320(21.323) |  |
| T3 | 177.206(22.355) | 152.695(28.277) |  |
| T4 | 191.959(33.335) | 142.715(42.165) |  |
| T5 | 314.406(46.878) | 330.949(59.297) |  |
| PaO_2_/FiO_2_ | | | |
| T1 | 398.475(31.624) | 346.960(40.002) | Group: 0.543  Time: <0.001  Group*Time: 0.512 |
| T2 | 331.925(35.501) | 255.013(44.906) |  |
| T3 | 238.204(29.126) | 253.030(36.841) |  |
| T4 | 272.437(31.284) | 294.290(39.572) |  |
| T5 | 177.222(29.532) | 165.760(37.356) |  |

a/A, arterial O_2_ partial pressure to alveolar O2 partial pressure ratio; A-a, alveolar-arterial; PaO_2_, partial pressure of oxygen; FiO_2_, fraction of inspired oxygen; T1, post-induction; T2, ACC 1h; T3, ACC 6h; T4, ACC 12h; T5, ACC 24h; Group, P value between the group; Time, P value of changes in the same group; Group*Time, P value of time-group interaction;

*** Cases combined with aortic root procedure which needed re-implantation of coronary arteries were not included in this subgroup analysis.
